# Associations Between Transdiagnostic Psychological Processes and Global Symptom Severity Among Outpatients With Various Mental Disorders: A Cross‐Sectional Study

**DOI:** 10.1002/cpp.70046

**Published:** 2025-02-07

**Authors:** Anna Katharina Frei, Thomas Studnitz, Britta Seiffer, Jana Welkerling, Johanna‐Marie Zeibig, Eva Herzog, Mia Maria Günak, Thomas Ehring, Keisuke Takano, Tristan Nakagawa, Leonie Sundmacher, Sebastian Himmler, Stefan Peters, Anna Lena Flagmeier, Lena Zwanzleitner, Ander Ramos‐Murguialday, Sebastian Wolf

**Affiliations:** ^1^ Faculty of Economics and Social Sciences, Institute of Sports Science, Department of Education & Health Research University of Tübingen Tübingen Germany; ^2^ Department of Psychology, Clinical Psychology and Psychological Treatment Ludwig‐Maximilians‐Universität München Munich Germany; ^3^ German Center for Mental Health (DZPG) Munich Germany; ^4^ Human Informatics and Interaction Research Institute The National Institute of Advanced Industrial Science and Technology (AIST) Tsukuba Japan; ^5^ Chair of Health Economics Technical University Munich (TUM) Munich Germany; ^6^ German Association for Health‐Related Fitness and Exercise Therapy (German: DVGS) Hürth‐Efferen Germany; ^7^ Department of Human Sciences, Institute of Sport Science Bundeswehr University Munich Munich Germany; ^8^ AOK Baden‐Württemberg Stuttgart Germany; ^9^ Techniker Krankenkasse Hamburg Germany; ^10^ Medical Faculty, Institute of Medical Psychology and Behavioral Neurobiology University of Tübingen Tübingen Germany; ^11^ Department of Neurology & Stroke University of Tübingen Tübingen Germany; ^12^ Tecnalia, Basque Research and Technology Alliance San Sebastián Spain; ^13^ Athenea Neuroclinics San Sebastián Spain

**Keywords:** emotion regulation, global symptom severity, mental disorders, outpatients, transdiagnostic psychological processes

## Abstract

**Objective:**

Knowledge about transdiagnostic factors associated with global symptom severity among patients diagnosed with various mental disorders remains limited. This study examined the cross‐sectional associations between transdiagnostic processes including global emotion regulation and specific emotion regulation strategies (i.e., amount of physical activity and sedentary behaviour, repetitive negative thinking and sleep routines) with global symptom severity, while controlling for sociodemographic data (age, gender, employment status, relationship status, and educational level) and fear of the coronavirus.

**Methods:**

Data from 401 outpatients, aged 42.08 years on average (*SD* = 13.26; 71.3% female), diagnosed with depressive disorders, non‐organic primary insomnia, agoraphobia, panic disorder and/or post‐traumatic stress disorder were examined. This study is a secondary analysis of a randomized controlled trial. Data were collected from 10 different study sites between March 2021 and May 2022 for cross‐sectional analysis. The influence of predictors of global symptom severity was determined using three‐step hierarchical multiple regression: (1) control variables, (2) global emotion regulation and (3) specific emotion regulation strategies. Global symptom severity was measured using the Global Severity Index, derived from the Brief Symptom Inventory‐18. Predictors were measured using validated scales, and physical activity was additionally assessed via accelerometer‐based sensors.

**Results:**

In the first step, control variables accounted for 4% of variance in global symptom severity. The inclusion of global emotion regulation in the second step explained 26% of the outcome variance, and the incorporation of specific emotion regulation strategies in the third step increased the explained variance to 37%. Significant predictors included global emotion regulation (*β* = 0.28), repetitive negative thinking (*β* = 0.26) and sleep routines (*β* = 0.25).

**Conclusion:**

Global emotion regulation along with repetitive negative thinking and sleep routines as specific emotion regulation strategies are identified as transdiagnostic psychological processes that may serve as treatment targets for evidence‐based interventions designed to enhance emotion regulation, particularly in transdiagnostic samples of stress‐related disorders. Additional prospective longitudinal studies with transdiagnostic samples are necessary to explore possible causal relationships.

**Summary**

- Global emotion regulation along with repetitive negative thinking and sleep routines as specific emotion regulation strategies can be considered transdiagnostic psychological processes associated with global symptom severity among outpatients diagnosed with various mental disorders.
- Treatment approaches aiming at improving transdiagnostic predictors of various mental disorders might be an efficacious target in reducing global symptom severity.
- Among outpatients (75% already receiving psychological or pharmacological treatment) diagnosed with depressive disorders, non‐organic primary insomnia, agoraphobia, panic disorder and/or PTSD, sociodemographic factors appear not to be predictive for global symptom severity.

## 1 Introduction

Major depressive disorder, anxiety disorders (such as panic disorder and agoraphobia), post‐traumatic stress disorder (PTSD) and non‐organic primary insomnia exhibit high comorbidity (Kessler et al. 2005; McGrath et al. 2020) and are characterized by partial symptom overlap (e.g., sleep problems) (American Psychiatric Association 2022). Several factors have been found that influence these mental disorders by posing a risk for their onset, predicting their symptom severity and/or contributing to their maintenance. Traditionally, variables involved in the development and maintenance of psychopathology have typically been studied from a disorder‐specific perspective; however, there is increasing evidence that transdiagnostic processes play an important role across different diagnostic categories (Dalgleish et al. 2020).

Depression, anxiety disorders, PTSD and insomnia are often referred to as ‘stress‐related’ disorders (Palagini et al. 2023; Smoller 2016), as severe acute stressful life events or prolonged exposure to stressors are risk factors for the development and exacerbation of psychiatric symptoms (Healey et al. 1981; Kessler 1997; Moreno‐Peral et al. 2014; Schwarzer and Luszczynska 2012). The COVID‐19 pandemic characterized by high mortality rates, isolation and overwhelmed healthcare systems can be considered such stressful life event, or rather period, and elevated stress levels during that time were reported (Gamonal‐Limcaoco et al. 2022). Indeed, meta‐analytic results suggest that fear of the coronavirus is related to anxiety, traumatic stress, depression and insomnia (Simsir et al. 2022). Stress can be defined as a mismatch between environmental or internal demands and an individual's adaptive resources such as coping (Monat and Lazarus 1991). Although demands are often uncontrollable and beyond our influence, coping strategies can be modified and learned. Emotion regulation is considered a coping strategy for stress by managing the emotional responses to stressful situations (Lazarus and Folkman 1984). It involves being aware of, understanding and accepting emotions and maintaining goal‐directed behaviour, while resisting impulsive actions during negative emotional states, along with having access to effective emotion regulation strategies. The Difficulties in Emotion Regulation Scale (DERS) (Gratz and Roemer 2004) captures these various facets of emotion regulation, of which some are prerequisites for emotional coping. For this reason, it serves as a comprehensive measure of emotion regulation. The Dynamic Fit Model (Myruski, Denefrio, and Dennis‐Tiwary 2020) suggests, however, that emotion regulation is an effective stress‐coping strategy only when there is a good match between the demands and the available emotion regulation resources. In fact, the ability to flexibly use emotion regulation strategies within and across different situational contexts is the strongest predictor of psychological functioning (Bonanno and Burton 2013). Deficits in emotion regulation, on the other hand, favour the development of depression (Berking et al. 2014), and among patients suffering from depression, these deficits are seen as one key factor in the maintenance of symptoms (Ehring et al. 2010). Impaired emotion regulation also appears to be significantly correlated with higher symptom severity of PTSD (Ehring and Quack 2010). Consequently, having access to a broad selection of emotion regulation strategies seems particularly beneficial; however, not all strategies individuals rely on are adaptive.

Physical activity can serve as an effective overt and adaptive emotion regulation strategy (Aldao and Dixon‐Gordon 2014), as it helps mitigate the negative effects of stress in daily life (Hachenberger et al. 2023). Additionally, it has been identified as an important lifestyle variable related to psychological well‐being among people with mental disorders (Firth et al. 2020). In fact, individuals who use physical activity as a strategy for affect regulation showed fewer psychiatric symptoms when being physically active compared with inactive individuals (Rosel et al. 2022). Furthermore, in people with mental disorders, those who show higher levels of daily physical activity are more likely to have better global functioning (Derhon et al. 2023). However, there is a lack of studies examining the impact of daily physical activity on mental health and meta‐analytic findings suggest that leisure‐time physical activity is related to positive mental health outcomes, but the associations with other types of activity, such as transportation or household chores, remain inconsistent (White et al. 2017).

Meta‐analyses further indicate that suffering from mental disorders is associated with elevated levels of sedentary behaviour (Schuch et al. 2017; van den Berk‐Clark et al. 2018; Vancampfort et al. 2017). Importantly, sedentary behaviour is associated with an increased risk of depression (Huang et al. 2020), anxiety (i.e., any anxiety disorder or anxiety symptoms) (Allen, Walter, and Swann 2019) and insomnia (Yang et al. 2017). Specifically, screen time–based sedentary behaviour is also associated with a heightened risk of depression (Wang, Li, and Fan 2019), and emerging research suggests that the problematic use of smartphones (Squires et al. 2021), excessive video gaming (Hollett and Harris 2020) and binge‐watching television (Alfonsi et al. 2023) can represent dysfunctional emotion regulation strategies.

Repetitive negative thinking is typically conceptualized as a maladaptive coping strategy for stress as it tends to impede effective problem‐solving abilities (Ward et al. 2003). Additionally, in both the general population and outpatients undergoing psychological treatment, enduring difficulties in emotion regulation may be explained by a tendency towards repetitive negative thinking (Mansueto et al. 2022). Rumination, a subtype of repetitive negative thinking, demonstrated the strongest association with psychopathology compared with other emotion regulation strategies such as reappraisal or avoidance (Aldao, Nolen‐Hoeksema, and Schweizer 2010). Importantly, repetitive negative thinking has been found to be present in a range of mental disorders, including depression, PTSD, insomnia and anxiety disorders (Ehring and Watkins 2008), and to be a risk factor for higher severity and maintenance of depressive and anxiety‐related symptoms (Spinhoven, van Hemert, and Penninx 2018). In addition, a systematic review about rumination concludes that it predicts PTSD symptom severity among patients diagnosed with PTSD (Moulds et al. 2020).

Furthermore, poorer sleep is associated with increased feelings of stress in individuals with sleep disorders (Demichelis et al. 2022), whereas adequate sleep is essential for managing stress effectively (Hamilton, Catley, and Karlson 2007). Importantly, sleep disturbances are not only part of the symptom criteria for many different disorders (e.g., depression and PTSD) but are also associated with higher levels of psychiatric symptom severity among patients with mood‐ and anxiety‐related disorders and PTSD (Belleville, Guay, and Marchand 2009; Kallestad et al. 2012). In contrast, good sleep hygiene practices are associated with increased use of adaptive emotion regulation strategies and decreased reliance on maladaptive ones (Parsons et al. 2022). Conversely, sleep deprivation may diminish the effectiveness of adaptive emotion regulation strategies (Zhang, Lau, and Hsiao 2019). Therefore, establishing and maintaining appropriate sleep routines or implementing sleep hygiene practices may serve as an effective emotion regulation strategy.

Given that these variables pose a risk for the onset of highly comorbid disorders such as depression, anxiety disorders, insomnia and PTSD, predict their symptom severity, contribute to their maintenance and share their associations with stress and their role as specific emotion regulation strategies, they might be regarded as transdiagnostic factors. This definition of transdiagnostic factors is consistent with criteria proposed in the literature (Dalgleish et al. 2020; Sauer‐Zavala et al. 2017). The so‐called mechanistically transdiagnostic constructs reveal core vulnerabilities across different mental disorders and provide insight into common mechanisms that might underly psychiatric symptoms (Harvey et al. 2011). Even though research about transdiagnostic approaches is growing, there is a scarcity of studies that assess the mentioned variables in transdiagnostic samples. In addition, only a small number of studies have conducted such assessments by simultaneously evaluating various predictors to determine their individual, distinct effects and to control for potential interactions. Furthermore, there are few studies that have examined this topic using a transdiagnostic outcome (e.g., global symptom severity). To address this research gap, we conducted a cross‐sectional assessment of the association between transdiagnostic factors and global symptom severity. First, we controlled for sociodemographic data (i.e., age, gender, employment status, relationship status and highest level of education) and fear of the coronavirus. Second, we incorporated global emotion regulation as a predictor to examine the impact of this overarching global construct. Third, we included the specific emotion regulation strategies (i.e., amount of sedentary behaviour and physical activity, interaction of physical activity and physical activity–related affect regulation, repetitive negative thinking and sleep routines) as a batch of additional predictors. This exploration could provide crucial insights and potentially initiate the development or refinement of transdiagnostic treatment approaches aimed at enhancing emotion regulation. These approaches would focus on those predictor variables that demonstrate a strong and significant association with global symptom severity within a sample of various mental disorders.

We tested the following hypothesis: In a sample of German outpatients diagnosed with depression, insomnia, panic disorder, agoraphobia and/or PTSD, global emotion regulation and specific emotion regulation strategies represent significant predictors of global symptom severity. It is further assumed that the specific emotion regulation strategies will provide incremental validity beyond global emotion regulation alone.

## 2 Methods

### 2.1 Study Design

This report is based on cross‐sectional data of the baseline assessment of the randomized controlled trial (RCT) ImPuls (Wolf et al. 2021; Wolf et al. 2024), which is an exercise intervention trial aimed at assessing the therapeutic impact of exercise. The ImPuls study was conducted according to the guidelines of the Declaration of Helsinki of 2010 and was approved by local ethics committee for medical research at the University of Tübingen (ID: 888/2020B01, 02/11/2020). The study was registered in the German Clinical Trial Register (ID: DRKS00024152, 05/02/2021). The current analysis was preregistered on Open Science Framework (https://osf.io/jhguz) before access to the cross‐sectional data was distributed on 21 March 2023. The preregistration was amended as it was necessary to exclude health‐related quality of life, perceived stress and emotional intelligence from the analysis (see explanation in Data S1).

### 2.2 Participants

Participants were recruited through inpatient psychiatric departments, general practitioners, psychiatric and psychotherapeutic outpatient units, (social) media and two major German health insurances [Allgemeine Ortskrankenkasse Baden‐Württemberg (AOK BW) and Techniker Krankenkasse (TK)]. Participants were included if the following inclusion criteria were fulfilled: age between 18 and 65 years, fluent in German, membership of the insurance companies AOK BW or TK, no medical contraindications for exercise (participants needed to confirm their ability to exercise through a medical consultation prior to the intervention) and at least one of the following diagnoses according to the International Classification of Diseases (10th ed.) (World Health Organization 2004): major depressive disorders (F32.1, F32.2, F33.1, F33.2), insomnia (F51.0), panic disorder (F41.0), agoraphobia (F40.0, F40.01) and PTSD (F43.1). Exclusion criteria were acute mental and behavioural disorders due to psychotropic substances (F10.0, F10.2–F10.9; F11.0, F11.2–F11.9; F12.0, F12.2–F12.9; F13.0, F13.2–F13.9; F14.0, F14.2–F14.9; F15.0, F15.2–F15.9; F16.0, F16.2–F16.9; F17.2–F17.9; F18.0, F18.2–F18.9; F19.0, F19.2–F19.9), acute eating disorders (F50), acute bipolar disorder (F31), acute schizophrenia (F20), acute suicidality, medical contraindication to exercise determined by a general practitioner or a specialized medical professional and regular engagement in at least moderate‐intensity exercise for at least 30 min, more than once a week, continuously over a 6‐week period within the last 3 months prior to study diagnosis. To investigate the therapeutic impact of exercise, it was necessary to exclude participants who were already physically active, as ceiling effects were expected.

The statistical power analysis for this report was calculated using G*Power, version 3.1.9.4 (Faul et al. 2009; Faul et al. 2007). With a proposed minimal effect of *f* ^2^ = 0.15 (Cohen 1988), number of predictors = 22, power = 0.80 and *α* = 0.05, the calculation resulted in a required minimum sample size of *n* = 163 for the current analysis.

**FIGURE 1:**
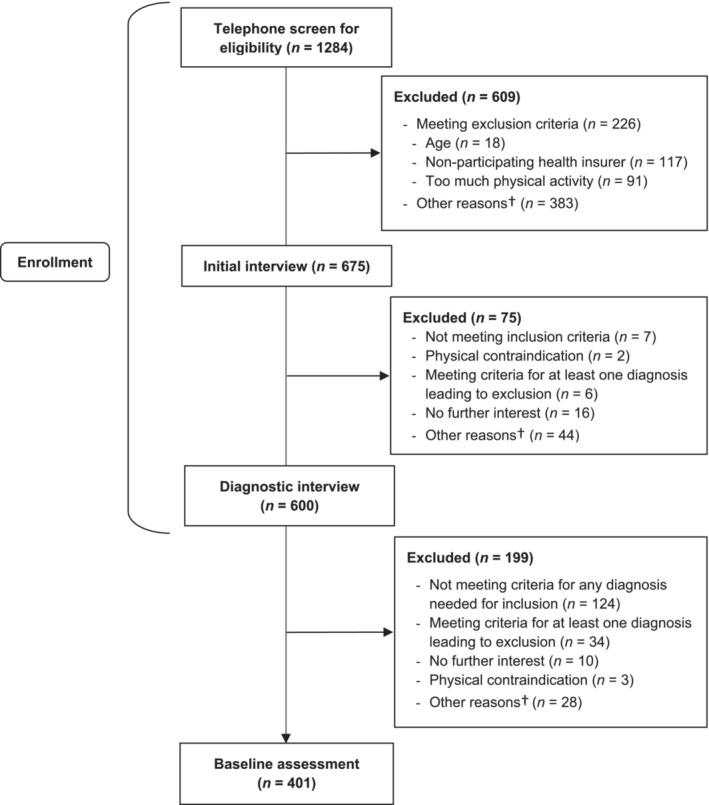
Flow of the participants up to the baseline assessment that is included in the current report. *Note:* ^†^Other reasons include organizational problems, relocation, no more contact possible, physical constraints, language problems and unknown.

Out of 1284 individuals who were screened for eligibility, 600 provided informed consent and underwent the structural diagnostic interview. Among these, 199 were excluded based on the inclusion and exclusion diagnoses, resulting in 401 individuals to be included in the cross‐sectional analysis. Figure 1 illustrates the flow of participants. Baseline demographic and clinical characteristics of the sample are shown in Table 1. The mean age was 42.08 years (*SD* = 13.26, range = 19–65) and 71.32% identified as female. Based on the Structured Clinical Interview (Beesdo‐Baum, Zaudig, and Wittchen 2019) according to the Diagnostic and Statistical Manual of Mental Disorders (5th ed.; DSM‐5) (American Psychiatric Association 2022), 72.1% of participants were diagnosed with depression (*n* = 289), 11.5% with panic disorder (*n* = 46), 9.2% with agoraphobia (*n* = 37), 18.0% with PTSD (*n* = 72) and 20.2% with primary insomnia (*n* = 81). At least one other inclusion diagnosis or another (nonexclusion) psychiatric diagnosis was present in 241 participants (60.1%). The detailed comorbid diagnoses included in the study are presented in Data S2. The mean global symptom severity at baseline assessment (*M* = 22.03, *SD* = 11.11) was comparable with the German clinical norm sample (*M* = 20.23, *SD* = 12.19) (Franke 2017).

**TABLE 1:** Baseline demographic and clinical characteristics of participants.

	*N* = 401		
	*n* (%)	*M* (*SD*)	Missing *n* (%)
Age (years)		42.08 (13.26)	4 (1.0)
Gender			0
Male	106 (26.4)		
Female	286 (71.3)		
Diverse	9 (2.2)		
Employment status			4 (1.0)
Unemployed	132 (32.9)		
Employed	223 (55.6)		
Other	42 (10.5)		
Relationship status			0
Single	180 (44.9)		
In relationship	221 (55.1)		
Highest level of education			0
None	1 (0.4)		
Primary	0		
Basic or intermediate secondary	99 (24.7)		
Vocational	25 (6.2)		
Secondary qualifying for university admission	131 (32.7)		
University	137 (34.2)		
Other	8 (2.0)		
Fear of the coronavirus (PAS)		11.12 (5.67)	4 (1.0)
Current treatment			
Outpatient psychological treatment^a^	215 (53.6)		85 (21.2)
Outpatient pharmacological treatment	215 (53.6)		2 (0.5)
Outpatient psychological or pharmacological treatment	301 (75.1)		58 (14.5)
Primary outcome			
Global Severity Index (BSI‐18)		22.03 (11.11)	1 (0.3)
Predictors			
Emotion regulation (DERS)		107.48 (23.80)	5 (1.3)
Physical activity, accelerometer‐measured			
Minutes/week spent in moderate to vigorous PA		335.45 (220.79)	46 (11.5)
Minutes/week spent in light PA		463.49 (198.90)	46 (11.5)
Minutes/week spent in sedentary behaviour		4835.33 (781.88)	46 (11.5)
Physical activity (minutes/week), self‐report (BSA‐F)		400.10 (640.72)	18 (4.5)
Physical activity‐related affect regulation (PA‐AR)		2.04 (0.75)	3 (0.8)
Repetitive negative thinking (PTQ)		39.11 (11.08)	5 (1.3)
Sleep quality (PSQI)		9.97 (3.81)	20 (5.0)
Primary diagnosis			
Depression (F32.1, F32.2, F33.1, F33.2)	289 (72.1)		0
Panic disorder (F41.0)	46 (11.5)		0
Agoraphobia (F40.00, F40.01)	37 (9.2)		0
Posttraumatic stress disorder (F43.1)	72 (18.0)		0
Primary insomnia (F51.0)	81 (20.2)		0
Comorbidity			
Any other inclusion diagnosis	98 (24.4)		0
Any other non‐inclusion psychiatric diagnosis	197 (49.1)		0
Any other inclusion diagnosis or any further psychiatric diagnosis	241 (60.1)		0

*Note:* Physical activity (PA; in minutes/week) assessed through accelerometer data and categorized by the metabolic equivalent of tasks (MET) into sedentary behaviour (SB; < 2.0 MET), light PA (LPA; 2.0–2.9 MET) and moderate to vigorous PA (MVPA; ≥ 3.0 MET) (Garber et al. 2011) or self‐report via the Physical Activity Index and Exercise Index of the Physical Activity, Exercise, and Sport Questionnaire (BSA‐F) (Fuchs et al. 2015); PA‐AR = PA‐related affect regulation assessed with the corresponding subscale of the PA‐related health competence questionnaire (Sudeck and Pfeifer 2016).

Abbreviations: DERS = Difficulties in Emotion Regulation Scale (Gratz and Roemer 2004); PAS = Pandemic Anxiety Scale (McElroy et al. 2020); PSQI = Pittsburgh Sleep Quality Index (Buysse et al. 1989); PTQ = Perseverative Thinking Questionnaire (Ehring et al. 2011).

^a^ An error in the questionnaire regarding current psychological treatment has resulted in a high number of missing responses.

### 2.3 Procedure

Data were collected at 10 different study sites in Baden‐Württemberg, Germany. The active enrolment period lasted from March 2021 until May 2022. Interested participants first attended a preliminary phone contact where they received general project information and completed screenings of eligibility criteria and somatic contraindications for exercise. Potentially eligible participants were then invited to an inhouse meeting taking place in the study site closest to their residence. They were informed about study procedures, provided written informed consent and were screened initially for exclusion diagnoses to prepare for the following Structured Clinical Interview for DSM‐5 Disorders (American Psychiatric Association 2022; Beesdo‐Baum, Zaudig, and Wittchen 2019) with a trained study psychologist. Once six participants at one study site were eligible for participation, they received online questionnaires via the web‐based data management system REDCap (P. A. Harris et al. 2019; P. A. Harris et al. 2009), which could be answered within a 14‐day period. Additionally, they wore accelerometer‐based physical activity sensors (MOVE 4; movisens GmbH) for seven consecutive days.

### 2.4 Outcomes

#### 2.4.1 Primary Outcome

Global symptom severity served as the primary outcome and was measured using the Global Severity Index (GSI), derived from the German version of the Brief Symptom Inventory‐18 [BSI‐18] (Franke 2017). The GSI encompasses ratings of general mental distress across somatization, depression and anxiety symptom scales. Each symptom scale consists of six items, resulting in a total of 18 items that were rated on a 5‐point Likert scale (range: 0–4). The total score for each scale is calculated, and the GSI is obtained by summing these three scores. Higher scores on the GSI indicate greater levels of distress, with a clinical cut‐off set at 12. Among patients with various mental disorders, the GSI has demonstrated good internal consistency (*α* = 0.89) (Franke 2017) and strong convergent validity (Franke et al. 2017; Spitzer et al. 2011). Cronbach's alpha of the GSI at baseline assessment in our study was *α* = 0.86, indicating good internal consistency.

#### 2.4.2 Predictor and Control Variables

Sociodemographic data as age, gender (i.e., female, male and diverse), employment status (i.e., employed, unemployed and other), relationship status (i.e., single and in relationship) and highest level of education (i.e., none, primary, secondary, vocational, high school diploma, university and other) were assessed via self‐report as part of the demographic questionnaire administered at the same measurement as the primary outcome and the other predictors.

Fear of the coronavirus was assessed with the German version of the Pandemic Anxiety Scale (PAS) (McElroy et al. 2020). The PAS exists of seven items forming a total score and assessing the subscales Disease Anxiety and Consequence Anxiety. The item scores range from 0 to 4, and higher scores indicate greater pandemic anxiety. In a general adult population sample, the PAS showed strong evidence of convergent and discriminant validity. Furthermore, in terms of predictive validity, the subscale Consequence Anxiety represented the strongest predictor of depression (Vargová et al. 2024).

Emotion regulation was assessed with the German version of the DERS (Gratz and Roemer 2004) that consists of 36 items. The total score is calculated by adding the scores of the items (range: 1–5). Higher scores indicate greater problems with emotion regulation. Among inpatients with severe mental illness, the DERS showed good construct validity by comparison with the Acceptance and Action Questionnaire‐II (Bond et al. 2011).

Accelerometer‐measured physical activity was assessed via accelerometer‐based sensors (Move 4, movisens GmbH). The sensor assesses physical activity based on kinematic data in three dimensions and atmospheric air pressure. This allows us to estimate the amount of physical activity through step counts and of different intensities for a specified time period based on validated algorithms (Anastasopoulou et al. 2012). Participants wore the sensors for seven consecutive days on the right hip. Physical activity was defined as any activity that exceeds 1.99 metabolic equivalents of tasks (MET), with light‐intensity physical activity requiring 2.0–2.9 METs and moderate to vigorous physical activity ≥ 3.0 METs. Accordingly, sedentary behaviour was defined as any activity less than 2.0 MET (Garber et al. 2011). Data were included as average time spent in sedentary behaviour, light physical activity and moderate to vigorous physical activity in minutes per day. Self‐reported physical activity in minutes per week was assessed using the self‐report Physical Activity Index (including job transportation, walking, cycling, physically demanding care and housework) and the Exercise Index (including physical activity with the goal of improving fitness) of the Physical Activity, Exercise, and Sport Questionnaire (BSA questionnaire) (Fuchs et al. 2015). Participants specified the type, duration and frequency of physical activity and exercise in the last 4 weeks. The scores were combined to a total score of physical activity in minutes per week. The BSA questionnaire shows good convergent, predictive and construct validity (Fuchs et al. 2015). Physical activity–related affect regulation was assessed with the corresponding subscale of the German questionnaire physical activity–related health competence (Sudeck and Pfeifer 2016). The subscale consists of four items rated on a 4‐point Likert scale (range: 1–4). The total score was calculated by averaging over all items with higher scores indicating greater physical activity–related affect regulation. The questionnaire demonstrates good convergent and discriminant validity (Sudeck and Pfeifer 2016).

Repetitive negative thinking was assessed with the German version of the Perseverative Thinking Questionnaire (PTQ) (Ehring et al. 2011). The PTQ consists of 15 items evaluating the assumed process characteristics of repetitive negative thinking with three items each (repetitive, intrusive, difficult to disengage from, unproductive, capturing mental capacity). The items are rated on a 5‐point Likert scale (range: 0–4), and higher scores indicate greater repetitive negative thinking. The PTQ demonstrates significant convergent validity when compared with other established measures of repetitive negative thinking (e.g., the Response Styles Questionnaire; Kühner, Huffziger, and Nolen‐Hoeksema 2007) and substantial predictive validity for symptoms of depression and anxiety disorders (Ehring et al. 2011) in a clinical sample.

Sleep routines were assessed with the global sleep quality score of the German version of the Pittsburgh Sleep Quality Index (PSQI) (Buysse et al. 1989). The global sleep quality score is the sum of seven sleep component scores (range: 0–3), including subjective sleep quality, sleep latency, sleep duration, habitual sleep efficiency, sleep disturbances, use of sleeping medications and daytime dysfunction. Higher scores indicate worse sleep quality. Adequate sleep can be attained trough the implementation of effective sleep hygiene practices. Notably, the global sleep quality score of the PSQI demonstrated a strong association (*r* = 0.48, *p* < 0.001) with a measure for sleep hygiene (i.e., Sleep Hygiene Index) (Mastin, Bryson, and Corwyn 2006). Specifically, various components of the PSQI are reflected in sleep hygiene rules. For example, the component of sleep disturbances pertains to avoiding daytime naps and the consumption of alcohol or caffeine in the evening as part of sleep hygiene practices (Buysse et al. 1989; Stepanski and Wyatt 2003). The PSQI has shown acceptable internal consistency and validity (Hinz et al. 2017).

### 2.5 Statistical Analysis

Data preparation and analysis were carried out using R, version 4.3.3 (R Core Team 2024). The analytic code is available at https://osf.io/5rcuz/files/osfstorage.

Descriptive statistics were used to analyse sample characteristics and are reported in frequencies (*n*) and percentages (%) for categorical variables and in means (*M*) and standard deviations (*SD*) for continuous variables. Self‐reported physical activity was manually checked for plausibility for values greater than 2000 min per week for the Physical Activity Index as well as greater than 200 min per week for the Exercise Index. Given the inactive nature of our participants due to the exclusion of individuals who regularly engaged in at least moderate‐intensity exercise for at least 30 min, more than once a week, continuously over a 6‐week period within the last 3 months prior to study diagnosis, these cutoffs were selected a priori to account for potential input errors in the questionnaires that could result in implausible data. Altogether, 11 values were omitted because they appeared unrealistic: 10 from the Physical Activity Index and one from the Exercise Index. Accelerometer‐measured data were included if at least four consecutive days with at least 8 h of wearing time were recorded as recommended by current guidelines (Donaldson et al. 2016; Migueles et al. 2017). We computed a mean activity value of the valid days and multiplied the result by seven to obtain a measure of minutes per week. All values were visually checked for the normality of distribution. Accelerometer‐based moderate to vigorous physical activity, self‐reported physical activity and physical activity–related affect regulation were log‐transformed due to skewness of data.

Data analysis of the primary outcome was performed using hierarchical multiple regression analyses to investigate incremental validity. Three models were tested to address the research question: The first model controlled for sociodemographic data and fear of the coronavirus, the second model incorporated global emotion regulation as a predictor to examine the impact of this overarching global construct, and the third model included the specific emotion regulation strategies (i.e., amount of sedentary behaviour and physical activity, interaction of physical activity and physical activity–related affect regulation, repetitive negative thinking and sleep routines) as a batch of additional predictors. Global symptom severity served as the outcome in all three models. Statistical significance was defined as a *p‐*value < 0.05. Assumptions of multiple linear regression (i.e., linearity, normality of the residuals, homoscedasticity and multicollinearity) were visually inspected or verified by a statistical test (i.e., Variance Inflation Factor for multicollinearity with a threshold of > 10 indicating multicollinearity). Standardized regression coefficients were calculated. Incremental validity was established using pooling methods for likelihood‐ratio tests (Meng and Rubin 1992) derived from the R package *mitml* for comparison of nested statistical models obtained from multiply imputed data sets (Grund, Robitzsch, and Luedtke 2023; Meng and Rubin 1992). Potential outliers of all variables included in the analysis were identified through three measures: interquartile range, Leverage and Cook's Distance. Thus, data points that are more than 1.5 times the interquartile range away from the 25th and 75th percentile, with a leverage of greater than 3*k/401 (with *k* = 13, 14 and 23 for model 1, 2 and 3, respectively) (Cohen et al. 2002), and a Cook's distance of greater than 0.5, were considered as potential outliers. No participants were excluded based on Cook's distance. However, *n* = 15 individuals exceeded both the interquartile range and high leverage criteria, indicating their potential influence on the model, and were therefore excluded. As unrealistic values related to the physical activity variable were excluded a priori, the remaining exclusions can be attributed to the presence of several extreme predictor values, which are considered realistic and plausible in the current analysis. Three‐step hierarchical multiple regression analyses were calculated again as a sensitivity analysis.

## 3 Results

### 3.1 Missing Values

After data collection, we identified 2.8% of missing data at scale level with 372 of 401 participants having at least one missing (92.8%). We used (scale‐based) multiple imputation to create 10 imputed datasets that contained all variables included in the current report. All analyses were then conducted using the 10 imputed datasets, and results were pooled according to Rubin's rules (Rubin 1987).

### 3.2 Hierarchical Multiple Regression Analysis and Incremental Validity

The first model, including sociodemographic data and fear of the coronavirus as control variables and global symptom severity as the dependent variable (outcome), explained 3.9% of variance in the GSI (adjusted *R* ^2^ = 0.039). Age (*β* = −0.12, *p* = 0.022) was significantly associated with global symptom severity. The second model, further including global emotion regulation (predictor), explained 26.2% of variance in the GSI (adjusted *R* ^2^ = 0.262). Fear of the coronavirus (*β* = 0.15, *p* = 0.001) and global emotion regulation (*β* = 0.49, *p* < 0.001) were significantly associated with global symptom severity. The third model, additionally including the specific emotion regulation strategies as predictors, explained 36.7% of variance in the GSI (adjusted *R* ^2^ = 0.367). Fear of the coronavirus (*β* = 0.12, *p* = 0.006), global emotion regulation (*β* = 0.28, *p* < 0.001), repetitive negative thinking (*β* = 0.26, *p* < 0.001) and sleep routines (*β* = 0.25, *p* < 0.001) were significantly associated with global symptom severity. There were no significant associations between sociodemographic data, physical activity or sedentary behaviour and global symptom severity. The inclusion of global emotion regulation in the second model resulted in a significant improvement in model fit [*F*(1, 20,123.50) = 105.81, *p* < 0.001]. The adjusted *R* ^2^ increased from 0.039 in the first model to 0.262 in the second model, suggesting that emotion regulation explained an additional 22.3% of variance in the GSI. The further addition of the specific emotion regulation strategies in the third model revealed a significant improvement in model fit compared with the second model [*F*(10, 36,720.73) = 7.60, *p* < 0.001]. The adjusted *R* ^2^ increased to 0.367, indicating that the specific emotion regulation strategies explained an additional 10.5% of variance in the GSI. The full statistics and regression coefficients of all three models are presented in Table 2. Pearson's correlation matrix of global symptom severity and predictor variables are provided in Data S3.

**TABLE 2:** Hierarchical multiple regression for global symptom severity and incremental validity (*N* = 401).

Model	Predictor	*B* [95% CI]	*SE*	*β*	*t*	*p*
1	*Control variables*					
	Age (years)	−0.07 [−0.16, 0.02]	0.04	−0.12	−2.30	**0.022**
	Gender			0.04	0.74	0.461
	Male	*ref*				
	Female	1.47 [−1.00, 3.94]	1.26			
	Diverse	0.42 [−7.12, 7.96]	3.83			
	Employment status			0.00	−0.02	0.985
	Employed	*ref*				
	Unemployed	1.36 [−1.05, 3.77]	1.23			
	Other	−1.43 [−5.05, 2.20]	1.84			
	Relationship status			0.05	1.08	0.279
	Single	*ref*				
	In relationship	1.58 [−0.64, 3.80]	1.13			
	Highest level of education			−0.07	−1.36	0.173
	None	*ref*				
	Basic or intermediate secondary	−9.81 [−33.54, 13.91]	12.02			
	Vocational	−13.25 [−37.24, 10.75]	12.16			
	Secondary qualifying for university admission	−8.84 [−32.48, 14.81]	11.98			
	University	−11.79 [−35.48, 11.90]	12.00			
	Other	−11.50 [−36.33, 13.33]	12.59			
	Fear of coronavirus (PAS)	0.34 [0.14, 0.53]	0.10	0.18	3.58	**< 0.001**
		*F*(6, 394) = 3.67, *p* = 0.001, adj. *R* ^2^ = 0.039				

		***B* [95% CI]**	***SE***	***β***	***t***	***p***
2	*Control variables*					
	Age (years)	0.02 [−0.06, 0.10]	0.04	0.01	0.21	0.831
	Gender			0.04	0.93	0.350
	Male	*ref*				
	Female	1.50 [−0.68, 3.67]	1.11			
	Diverse	−0.14 [−6.77, 6.50]	3.37			
	Employment status			−0.01	−0.32	0.749
	Employed	*ref*				
	Unemployed	0.57 [−1.56, 2.70]	1.08			
	Other	−1.20 [−4.39, 1.99]	1.62			
	Relationship status			0.05	1.15	0.249
	Single	*ref*				
	In relationship	1.26 [−0.70, 3.21]	1.00			

	Highest level of education			−0.02	−0.34	0.732
	None	*ref*				
	Basic or intermediate secondary	−9.15 [−30.54, 12.24]	10.82			
	Vocational	−11.75 [−33.37, 9.87]	10.94			
	Secondary qualifying for university admission	−8.50 [−29.82, 12.82]	10.78			
	University	−9.52 [−30.88, 11.83]	10.80			
	Other	−10.02 [−32.35, 12.31]	11.30			
	Fear of coronavirus (PAS)	0.28 [0.11, 0.45]	0.09	0.15	3.41	**0.001**
	*Emotion regulation*					
Emotion regulation (DERS)	0.22 [0.18, 0.27]	0.02	0.49	10.93	**< 0.001**
		*F*(7, 393) = 21.09, *p* < 0.001, adj. *R* ^2^ = 0.262				
Incremental validity of Model 1 vs. Model 2		*F*(1, 20,123.50) = 105.81, *p* < 0.001, Δ adj. *R* ^2^ = 0.223				

		***B* [95% CI]**	***SE***	***β***	***t***	***p***
3	*Control variables*					
	Age (years)	0.01 [−0.07, 0.08]	0.04	0.00	−0.10	0.923
	Gender			0.01	0.28	0.779
	Male	*ref*				
	Female	0.52 [−1.72, 2.76]	1.14			
	Diverse	−0.37 [−6.72, 5.98]	3.23			
	Employment status			−0.02	−0.42	0.673
	Employed	*ref*				
	Unemployed	0.08 [−1.95, 2.11]	1.03			
	Other	−0.82 [−3.85, 2.22]	1.54			
	Relationship status			0.04	0.93	0.352
	Single	*ref*				
	In relationship	0.90 [−0.97, 2.76]	0.95			
	Highest level of education			−0.01	−0.31	0.756
	None	*ref*				
	Basic or intermediate secondary	−4.10 [−25.86, 17.65]	10.94			
	Vocational	−6.29 [−28.20, 15.61]	11.02			
	Secondary qualifying for university admission	−3.68 [−25.24, 17.87]	10.84			
	University	−4.34 [−25.89, 17.21]	10.84			
	Other	−6.52 [−28.95, 15.91]	11.30			
	Fear of coronavirus (PAS)	0.22 [0.05, 0.38]	0.08	0.12	2.77	**0.006**
	*Emotion regulation*					
	Emotion regulation (DERS)	0.13 [0.08, 0.18]	0.03	0.28	5.01	**< 0.001**

	*Emotion regulation strategies*					
	MVPA, accelerometer‐measured	−1.77 [−4.30, 0.76]	1.28	−0.02	−0.32	0.750
	LPA, accelerometer‐measured	0.00 [−0.01, 0.01]	0.01	−0.05	−0.99	0.321
	SB, accelerometer‐measured	0.00 [0.00, 0.00]	0.00	−0.02	−0.45	0.652
	PA, self‐reported (BSA‐F)	−1.08 [−2.65, 0.50]	0.80	−0.03	−0.62	0.537
	MVPA (accelerometer‐measured) × PA‐related affect regulation (PA‐AR)	2.32 [−1.12, 5.76]	1.75	0.06	1.40	0.162
	LPA (accelerometer‐measured) × PA‐related affect regulation (PA‐AR)	−0.01 [−0.02, 0.01]	0.01	−0.06	−1.16	0.247
	PA self‐reported (BSA‐F) × PA‐related affect regulation (PA‐AR)	1.24 [−1.02, 3.49]	1.15	0.04	1.03	0.305
	Repetitive negative thinking (PTQ)	0.26 [0.15, 0.36]	0.05	0.26	4.94	**< 0.001**
Sleep quality (PSQI)	0.74 [0.50, 0.99]	0.13	0.25	5.99	**< 0.001**
		*F*(17, 378) = 14.27, *p* < 0.001, adj. *R* ^2^ = 0.367				
Incremental validity of Model 2 vs. Model 3		*F*(10, 36,720.73) = 7.60, *p* < 0.001, Δ adj. *R* ^2^ = 0.105				

*Note:* Physical activity (PA; in minutes/week) assessed through accelerometer data and categorized by the metabolic equivalent of tasks (MET) into sedentary behaviour (SB; < 2.0 MET), light PA (LPA; 2.0–2.9 MET) and moderate to vigorous PA (MVPA; ≥ 3.0 MET) (Garber et al. 2011) or self‐report via the Physical Activity Index and Exercise Index of the Physical Activity, Exercise, and Sport Questionnaire (BSA‐F) (Fuchs et al. 2015); PA‐AR = PA‐related affect regulation assessed with the corresponding subscale of the PA‐related health competence questionnaire (Sudeck and Pfeifer 2016). B: pooled unstandardized regression coefficient, 95% CI: 95% confidence interval for B, SE: standard error, *β*: pooled standardized regression coefficient. Statistical significance: *p* < 0.05.

Abbreviations: DERS = Difficulties in Emotion Regulation Scale (Gratz and Roemer 2004); PAS = Pandemic Anxiety Scale (McElroy et al. 2020); PSQI = Pittsburgh Sleep Quality Index (Buysse et al. 1989); PTQ = Perseverative Thinking Questionnaire (Ehring et al. 2011).

### 3.3 Sensitivity Analysis

The sensitivity analysis after the exclusion of identified outliers revealed similar results that are provided in detail in Data S4.

## 4 Discussion

In this study, we examined cross‐sectional associations of transdiagnostic predictors (i.e., global emotion regulation and specific emotion regulation strategies) of global symptom severity among outpatients diagnosed with depressive disorders, non‐organic primary insomnia, agoraphobia, panic disorder and/or PTSD. Greater difficulties with global emotion regulation, along with higher levels of repetitive negative thinking and worse sleep routines/sleep quality—transdiagnostic psychological processes typically considered as emotion regulation strategies and involved in the adaption to stressful life events—were significantly associated with higher global symptom severity in our outpatient sample. This association was observed while controlling for sociodemographic data and fear of the coronavirus in a multiple regression analysis. Furthermore, incorporating specific emotion regulation strategies—repetitive negative thinking and sleep routines—into the model, which initially contained only control variables and a global measure of emotion regulation assessing emotional awareness, understanding and acceptance, as well as the flexible use of appropriate emotion regulation strategies, significantly increased the explained variance in global symptom severity. This addition also reduced the influence of global emotion regulation on global symptom severity by half. Thus, repetitive negative thinking and sleep routines function as specific emotion regulation strategies, and as such, they explain additional variance beyond what is accounted for by global emotion regulation alone. However, contrary to our hypothesis, no associations between global symptom severity and the amount of daily physical activity and sedentary behaviour were found. The sensitivity analysis after the exclusion of identified outliers also revealed comparable results. Hence, the results suggest that global emotion regulation, along with repetitive negative thinking and sleep routines as specific emotion regulation strategies, each represent important transdiagnostic factors in our transdiagnostic sample.

As for the control variables, heightened fear of the coronavirus was significantly associated with global symptom severity in all three models. During the active enrolment period, an average of 463 (*SD* = 589) confirmed new coronavirus infections per 100,000 inhabitants were reported in Baden‐Württemberg over a 7‐day period (infas 360 2024). The consequences associated with these high incidence rates, such as elevated mortality rates and overwhelmed healthcare systems, may help explain the significant association between increased fear of the coronavirus and higher levels of global symptom severity. Furthermore, younger age was significantly associated with higher global symptom severity in the first model, and this relationship appeared to be influenced by individuals' emotion regulation abilities. Specifically, when emotion regulation was incorporated into the analysis, the association between age and symptom severity disappeared, likely because younger individuals of our sample tend to struggle more with emotion regulation (see correlation matrix in Data S3), resulting in more severe symptoms in the initial model. In the second and third models, none of the sociodemographic data were significantly associated with global symptom severity over and above psychological variables. This finding suggests that in an outpatient sample already diagnosed with and suffering from mental disorders, primarily psychological variables account for variance on global symptom severity. Sociodemographic factors such as being female, a young adult, single, unemployed or retired and belonging to a lower social class seem to increase the likelihood of becoming prevalent with a mental disorder (Jacobi et al. 2014a; Jacobi et al. 2004) rather than explaining the variance of psychopathology within a sample of mentally ill patients. Thus, these sociodemographic factors are considered more distal and do not directly influence psychopathology; instead, their effects are mediated by more proximal processes, such as psychological factors. If the selection of proximal processes is sufficiently comprehensive, no direct effects of distal variables (i.e., sociodemographic data) are to be expected in this context. In addition, the finding that nonmodifiable sociodemographic variables show no significant association with global symptom severity, in contrast to modifiable psychological variables which do, and account for a significant amount of variance supports the idea of reducing global symptom severity in patients through transdiagnostic interventions.

Research highlights the key role of emotion regulation in the onset, maintenance and severity of the included mental disorders, making it a prime target for interventions. Indeed, strategies aimed at fostering emotional regulation are core components of many disorder‐specific psychological interventions, and emotion regulation is considered an important change mechanism of psychotherapy (Palmieri et al. 2022). Our findings align with existing research on emotion regulation as a transdiagnostic process and offer additional insights into specific emotion regulation strategies that may serve as a crucial foundation for the development of evidence‐based transdiagnostic interventions.

In the form of repetitive negative thinking, our study examined two commonly studied emotion regulation strategies, rumination and worry (Aldao, Nolen‐Hoeksema, and Schweizer 2010; Ehring et al. 2011; Naragon‐Gainey, McMahon, and Chacko 2017). Previous research supports the idea that rumination represents an important emotion regulation strategy influencing various symptoms (Aldao, Nolen‐Hoeksema, and Schweizer 2010), whereas worry is typically conceptualized as a strategy for coping with or regulating anxiety‐related emotions (Salters‐Pedneault et al. 2006). Although rumination and worry share several characteristics, such as being repetitive and perseverative, their potential regulatory functions differ. Rumination may be employed as an attempt to solve problems or negative experiences through constant contemplation. In contrast, excessive worrying may serve to distract from or suppress more intense negative emotions while preparing for potential adverse outcomes. However, rumination often appears to interfere with good problem‐solving, and worry tends to maintain or even exacerbate negative affect rather than alleviate it (Stapinski, Abbott, and Rapee 2010; Ward et al. 2003). Thus, our results extend existing literature and suggest that repetitive negative thinking might be a maladaptive transdiagnostic emotion regulation strategy in patients with various stress‐related disorders.

Our results further provide evidence supporting that good sleep routines can be regarded as an emotion regulation strategy, although existing literature on this topic remains limited. Most research has primarily focused on the emotional consequences of sleep deprivation (Palmer and Alfano 2017) or on how negative emotions can disrupt sleep quality (Krizan, Boehm, and Strauel 2024), rather than examining adequate sleep routines itself as a means of regulating emotions. Our findings support the notion that sleep routines serve as an emotion regulation strategy, as indicated by the significant correlation with global emotion regulation shown in Data S3. Notably, our study utilizes a transdiagnostic sample, further enriching this area of research.

Contrary to our hypothesis, no significant associations were found for either daily physical activity or sedentary behaviour and global symptom severity. This report is based on cross‐sectional data of the baseline assessment of an exercise intervention trial aimed at assessing the therapeutic impact of exercise. Therefore, individuals who had engaged in exercise for more than 30 min per week in the last 3 months before the study diagnosis were excluded, ensuring that only inactive patients participated. This specific exclusion criterion minimized the variance of the physical activity variable within the sample and the resulting loss of information might have led to the non‐significant association with global symptom severity. Additionally, the amount of physical activity analysed in this study primarily captured routine daily activities such as transportation, household chores, leisure‐time pursuits and occupational tasks. Although leisure‐time physical activity is associated with positive mental health outcomes, evidence suggests inconsistent or even negative associations for activities in occupational, transportation and domestic domains, with some linked to increased depressive symptoms or more experienced psychological distress (Asztalos et al. 2009; Lopes et al. 2023; White et al. 2017). One explanation is that these activities, often obligatory in nature, are rarely chosen for enjoyment or as a means to regulate affect, which may account for the lack of significant associations in our analysis.

Up to this point, these psychological factors have primarily been assessed in relation to specific mental disorders (Scott et al. 2021; Sloan et al. 2017; Wahl et al. 2019; Zorn et al. 2017). The findings of this study therefore represent an important expansion of existing literature by demonstrating that underlying mechanisms predominantly investigated in disorder‐specific samples also hold relevance in transdiagnostic contexts. The results suggest that global emotion regulation along with repetitive negative thinking and sleep routines as specific emotion regulation strategies might be core transdiagnostic factors that are present across various stress‐related mental disorders. From a theoretical perspective, these findings lend further support to transdiagnostic approaches. The potential clinical relevance lies in the identification of psychological factors that could serve as targets for transdiagnostic interventions aimed at enhancing emotion regulation abilities, specifically for stress‐associated disorders. Indeed, meta‐analytic evidence demonstrates that interventions targeting repetitive negative thinking—a key emotion regulation strategy—can effectively alleviate symptoms of stress‐related disorders, specifically anxiety and depression, in young people (Egan et al. 2024). This approach is further supported by a growing body of evidence showing that transdiagnostic cognitive behavioural therapy is not only as effective as disorder‐specific therapy but also superior to waitlist and treatment‐as‐usual approaches in addressing emotional disorders (Schaeuffele et al. 2024). Furthermore, repetitive negative thinking and sleep disturbances are associated with several clinically significant outcomes, including suicidal behaviour, reduced quality of life and impaired social and global functioning (Adamis et al. 2024; Caudle et al. 2024; L. M. Harris et al. 2020; Kallestad et al. 2012), highlighting their potential as transdiagnostic treatment targets. The results are particularly relevant given the limited number of studies exploring psychological predictors of transdiagnostic outcomes such as global symptom severity within transdiagnostic outpatient samples.

It is important to recognize that the actual sample size was more than twice as large as the minimum sample size required, determined by power calculation. A post hoc power calculation with the given sample size of 401 revealed a power of 99.9% for the current report (Faul et al. 2009; Faul et al. 2007). However, when interpreting the results of overpowered studies, statistical significance should not be confused with clinically meaningful effects (Sedgwick 2013). Therefore, it is crucial to consider the reported effect sizes and the degree of variance explanation, alongside mere *p*‐values. The effect sizes of our models, particularly of the third model, as well as the explained variance in global symptom severity can be regarded as acceptable in social science research given the complexity of human behaviour and the number of potential unmeasured factors. These results become even more convincing when considering that the majority of our sample received psychological or pharmacological treatment at the time of assessment, and still, the psychological factors were strongly and significantly associated with global symptom severity.

### 4.1 Strengths and Limitations

A key strength of this study comprises the large transdiagnostic sample consisting of German outpatients whose clinical diagnoses were verified through structured interviews conducted by trained study psychologists. The study sample further enables the generalization of results to the German population diagnosed with major depressive disorder, anxiety disorders, agoraphobia, panic disorder, PTSD and non‐organic primary insomnia. The mean age of our sample, 42.1 years, aligns with the mean age of the general population, which is 44.6 years (Federal Statistical Office 2023). Additionally, the majority of participants being female corresponds with the higher incidence of mental disorders, such as affective and anxiety disorders, among women. Notably, depression is considered more prevalent than the other diagnoses included in the study (Jacobi et al. 2014b; Jacobi et al. 2016). Furthermore, all predictors were measured by validated and widely used scales or accelerometer‐based sensors, and an external institution was responsible for data management, including data collection, storing and anonymization. In addition, participants were blinded to the research question, which minimized potential biases, and a wide range of potential predictors of global symptom severity were simultaneously investigated within a transdiagnostic sample. The study also faces several limitations due to the cross‐sectional research design, which makes it impossible to draw causal conclusions. It is crucial to conduct additional prospective and longitudinal studies for a more thorough exploration of the causal relationships involved. Furthermore, this study is a secondary cross‐sectional analysis of baseline data from an RCT evaluating the efficacy of the group‐based exercise intervention, ImPuls. Although the research question and selection of predictors were preregistered for this analysis, the inclusion and exclusion criteria, along with the predictors, were primarily established during the RCT's conceptualization. This may limit the generalizability of our findings, as the study is restricted to a specific selection of stress‐related disorders and predictors. In addition, the sample was restricted as only physically inactive individuals were included, that is, participants who had exercised continuously for at least 30 min at least twice a week in the last 3 months before the study diagnosis were excluded. This criterion particularly limited leisure‐time physical activity, which is the domain typically associated with mental health. Excluding physically active individuals restricts the generalizability of our results regarding physical activity and may have contributed to the lack of significant associations with global symptom severity. Lastly, we only included patients from two German health insurance companies. Although they cover a very large proportion of insured people, this still limits the representativeness of our sample.

### 4.2 Conclusion and Future Directions

This report examined cross‐sectional associations of emotion regulation and specific emotion regulation strategies with global symptom severity among outpatients with various stress‐related mental disorders. The findings highlight that common and prevalent psychological processes as emotion regulation, along with repetitive negative thinking and sleep routines as specific emotion regulation strategies, each have a significant association with global transdiagnostic symptom severity among patients diagnosed with depressive disorders, non‐organic primary insomnia, agoraphobia, panic disorder and/or PTSD. Thus, emotion regulation strategies predominantly studied in disorder‐specific samples also hold relevance in transdiagnostic contexts. Consequently, these emotion regulation strategies hold promise as targets not only in disorder‐specific interventions but also in transdiagnostic treatment approaches. However, further research in prospective longitudinal studies with transdiagnostic samples is warranted.

## Conflicts of Interest

The authors declare no conflicts of interest.

## Supporting information


**Data S1** Supplementary Information.

**Data S2** Supplementary Information.

**Data S3** Supplementary Information.

**Data S4** Supplementary Information.

## Data Availability

The data that support the findings of this study are openly available in Open Science Framework at https://osf.io/5rcuz/files/osfstorage
