# Supplementary material for: Associations Between Transdiagnostic Psychological Processes and Global Symptom Severity Among Outpatients With Various Mental Disorders: A Cross‐Sectional Study
Source: Clin Psychol Psychother. 2025 Feb 7;32(1):e70046. doi: 10.1002/cpp.70046 (PMC11803435; doi:10.1002/cpp.70046)
Supplement: Supplementary file 1 — Data S1 Supplementary Information. [file CPP-32-e70046-s003.docx]

**S1. Deviation from preregistration**

We excluded health-related quality of life from our analysis as this variable does not align with our definition of an impact factor. In addition, a correlation analysis of the EQ-5D-5L questionnaire ([Herdman et al., 2011](#_ENREF_2); [Hinz, Kohlmann, Stobel-Richter, Zenger, & Brahler, 2014](#_ENREF_3)) with the primary outcome (i.e., global symptom severity measured using the Global Severity Index of the Brief Symptom Inventory [BSI-18] ([Franke, 2017](#_ENREF_1)) demonstrated a high correlation (*r* = -.608). This strong correlation may be attributed to the fact that the domain "anxiety or depression" of the EQ-5D-5L questionnaire corresponds to the depression and anxiety subscales of the BSI-18 ([Franke, 2017](#_ENREF_1)). Additionally, during the review process we further excluded emotional intelligence and perceived stress as predictors from our analysis. Emotional intelligence does not align with our conceptualization of a transdiagnostic psychological process, as it is regarded as a trait ([Petrides, Pita, & Kokkinaki, 2007](#_ENREF_4)). Similarly, perceived stress is better understood as a correlate of symptomatology and is related with the other predictors rather than representing an emotion regulation strategy.

References

Franke, G. H. (2017). *Mini-Symptom-Checklist* (Vol. 1). Göttingen: Hogrefe.

Herdman, M., Gudex, C., Lloyd, A., Janssen, M., Kind, P., Parkin, D., . . . Badia, X. (2011). Development and preliminary testing of the new five-level version of EQ-5D (EQ-5D-5L). *Quality of Life Research, 20*(10), 1727-1736. doi:10.1007/s11136-011-9903-x

Hinz, A., Kohlmann, T., Stobel-Richter, Y., Zenger, M., & Brahler, E. (2014). The quality of life questionnaire EQ-5D-5L: psychometric properties and normative values for the general German population. *Quality of Life Research, 23*(2), 443-447. doi:10.1007/s11136-013-0498-2

Petrides, K. V., Pita, R., & Kokkinaki, F. (2007). The location of trait emotional intelligence in personality factor space. *British Journal of Psychology, 98*(Pt 2), 273-289. doi:10.1348/000712606X120618
