# Supplementary material for: Associations Between Transdiagnostic Psychological Processes and Global Symptom Severity Among Outpatients With Various Mental Disorders: A Cross‐Sectional Study
Source: Clin Psychol Psychother. 2025 Feb 7;32(1):e70046. doi: 10.1002/cpp.70046 (PMC11803435; doi:10.1002/cpp.70046)
Supplement: Supplementary file 2 — Data S2 Supplementary Information. [file CPP-32-e70046-s002.docx]

**S2.** Comorbid diagnoses (*N* = 401)

|  | *n* (%) |
| --- | --- |
| Mental and behavioral disorders due to harmful use of alcohol (F10.1, F10.10) | 11 (2.7) |
| Mental and behavioral disorders due to harmful use of cannabinoids (F12.1, F12.10) | 5 (1.2) |
| Mild depressive disorder (F32.0, F32.4) | 11 (2.7) |
| Premenstrual dysphoric disorder (F32.81) | 10 (2.5) |
| Mild or partially remitted recurrent depressive disorder (F33.0, F33.4) | 21 (5.2) |
| Dysthymia (F34.1) | 83 (20.7) |
| Social phobia anxiety disorders (F40.1, F40.10) | 34 (8.5) |
| Specific (isolated) phobias (F40.2) | 29 (7.2) |
| Generalized anxiety disorder (41.1) | 14 (3.5) |
| Obsessive-compulsive disorders (F42.0, F42.1, F42.2, F42.3, F42.4) | 23 (5.7) |
| Somatoform disorders (F45.0, F45.1, F45.2, F45.41) | 15 (3.7) |
| Hyperkinetic disorders (F90.0, F90.1, F90.2) | 10 (2.5) |
