# Supplementary material for: Associations Between Transdiagnostic Psychological Processes and Global Symptom Severity Among Outpatients With Various Mental Disorders: A Cross‐Sectional Study
Source: Clin Psychol Psychother. 2025 Feb 7;32(1):e70046. doi: 10.1002/cpp.70046 (PMC11803435; doi:10.1002/cpp.70046)
Supplement: Supplementary file 3 — Data S3 Supplementary Information. [file CPP-32-e70046-s004.docx]

**S3.** Pearson’s correlation matrix of global symptom severity and potential predictors

| **Variables** | 1 | 2 | 3 | 4 | 5 | 6 | 7 | 8 |
| --- | --- | --- | --- | --- | --- | --- | --- | --- |
| 1. global symptom severity | 1 |  |  |  |  |  |  |  |
| 2. emotion regulation | 0.49*** | 1 |  |  |  |  |  |  |
| 3. MVPA, accelerometer-measured | -0.07 | -0.02 | 1 |  |  |  |  |  |
| 4. LPA, accelerometer-measured | -0.05 | -0.07 | 0.48** | 1 |  |  |  |  |
| 5. SB, accelerometer-measured | -0.04 | -0.03 | -0.35** | -0.27*** | 1 |  |  |  |
| 6. PA, self-reported | -0.08 | -0.14 | 0.21** | 0.23*** | -0.08 | 1 |  |  |
| 7. repetitive negative thinking | 0.48*** | 0.63*** | 0.08 | 0.05 | -0.12 | -0.06 | 1 |  |
| 8. sleep quality | 0.34*** | 0.16* | -0.13 | 0.05 | -0.01 | 0.05 | 0.12 | 1 |
| *Note.* MVPA = moderate to vigorous physical activity, accelerometer-measured; LPA = light physical activity, accelerometer-measured; SB = sedentary behavior, accelerometer-measured; PA = physical activity, self-reported.  For reasons of clarity, the bivariate correlations with the control variables (i.e., sociodemographic data and fear of the coronavirus) were excluded from this correlation matrix. Significant correlations were found for the following variables: Fear of the coronavirus was significantly correlated with global symptom severity (*r* = 0.18*); age was significantly correlated with emotion regulation (*r* = -0.23***), LPA (*r* = 0.19*), PA (*r* = 0.17*), and repetitive negative thinking (*r* = -0.18*).  ****p* < .001, ** *p* < .01, **p* < .05. | | | | | | | | |
