# Supplementary material for: Associations Between Transdiagnostic Psychological Processes and Global Symptom Severity Among Outpatients With Various Mental Disorders: A Cross‐Sectional Study
Source: Clin Psychol Psychother. 2025 Feb 7;32(1):e70046. doi: 10.1002/cpp.70046 (PMC11803435; doi:10.1002/cpp.70046)
Supplement: Supplementary file 4 — Data S4 Supplementary Information. [file CPP-32-e70046-s001.docx]

**S5.** Hierarchical multiple regression for global symptom severity and incremental validity after exclusion of identified outliers (*N* = 386)

| Model | Predictor | **B [95% CI]** | ***SE*** | **β** | ***t*** | ***p*** |
| --- | --- | --- | --- | --- | --- | --- |
| 1 | *Control variables* |  |  |  |  |  |
|  | **age (years)** | -0.09 [-0.18, 0.00] | 0.05 | -.13 | -2.49 | **.013** |
|  | **gender** |  |  | .03 | 0.63 | .531 |
|  | male | *ref* |  |  |  |  |
|  | female | 1.49 [-1.04, 4.02] | 1.29 |  |  |  |
|  | diverse | -0.30 [-8.26, 7.67] | 4.05 |  |  |  |
|  | **employment status** |  |  | -.01 | -0.16 | .875 |
|  | employed | *ref* |  |  |  |  |
|  | unemployed | 1.72 [-0.76, 4.19] | 1.26 |  |  |  |
|  | other | -2.23 [-6.07, 1.61] | 1.95 |  |  |  |
|  | **relationship status** |  |  | .05 | 1.07 | .286 |
|  | single | *ref* |  |  |  |  |
|  | in relationship | 1.58 [-0.69, 3.84] | 1.15 |  |  |  |
|  | **highest level of education** |  |  | -.07 | -1.28 | .201 |
|  | none | *ref* |  |  |  |  |
|  | basic or intermediate secondary | -10.37 [-34.53, 13.78] | 12.22 |  |  |  |
|  | vocational | -13.94 [-38.39, 10.51] | 12.37 |  |  |  |
|  | secondary qualifying for university admission | -9.50 [-33.58, 14.58] | 12.18 |  |  |  |
|  | university | -11.89 [-36.03, 12.26] | 12.21 |  |  |  |
|  | other | -13.68 [-39.11, 11.76] | 12.88 |  |  |  |
|  | fear of coronavirus (PAS) | 0.28 [0.09, 0.48] | 0.10 | .16 | 3.06 | **.002** |
|  |  | ***F*(6, 379) = 3.13, *p* = .005*, adj. R^2^* = 0.033** | | | | |
|  |  | **B [95% CI]** | ***SE*** | **β** | ***t*** | ***p*** |
| 2 | *Control variables* |  |  |  |  |  |
|  | **age (years)** | 0.00 [-0.08, 0.08] | 0.04 | .00 | -0.09 | .926 |
|  | **gender** |  |  | .04 | 0.80 | .422 |
|  | male | *ref* |  |  |  |  |
|  | female | 1.47 [-0.74, 3.69] | 1.13 |  |  |  |
|  | diverse | -0.70 [-7.67, 6.27] | 3.55 |  |  |  |
|  | **employment status** |  |  | -.02 | -0.51 | .610 |
|  | employed | *ref* |  |  |  |  |
|  | unemployed | 0.83 [-1.35, 3.00] | 1.10 |  |  |  |
|  | other | -1.95 [-5.32, 1.41] | 1.71 |  |  |  |
|  | **relationship status** |  |  | .05 | 1.02 | .307 |
|  | single | *ref* |  |  |  |  |
|  | in relationship | 1.13 [-0.86, 3.12] | 1.01 |  |  |  |
|  | **highest level of education** |  |  | -.01 | -0.25 | .806 |
|  | none | *ref* |  |  |  |  |
|  | basic or intermediate secondary | -9.76 [-31.52, 12.00] | 10.98 |  |  |  |
|  | vocational | -12.34 [-34.35, 9.66] | 11.11 |  |  |  |
|  | secondary qualifying for university admission | -9.18 [-30.88, 12.53] | 10.95 |  |  |  |
|  | university | -9.67 [-31.42, 12.09] | 10.98 |  |  |  |
|  | other | -12.17 [-35.01, 10.68] | 11.55 |  |  |  |
|  | fear of coronavirus (PAS) | 0.22 [0.05, 0.39] | 0.09 | .12 | 2.78 | **.006** |
|  | *emotion regulation* |  |  |  |  |  |
|  | emotion regulation (DERS) | 0.23 [0.19, 0.27] | 0.02 | .50 | 10.95 | **<.001** |
|  |  | ***F*(7, 378) = 20.58,  *p* < .001*, adj. R^2^* = 0.264** | | | | |
| Incremental validity of model 1 vs. model 2 | | ***F*(1, 27111.77) = 105.62, *p* < .001, *Δ adj. R^2^* = 0.231** | | | | |
|  |  | **B [95% CI]** | ***SE*** | **β** | ***t*** | ***p*** |
| 3 | *Control variables* |  |  |  |  |  |
|  | **age (years)** | -0.01 [-0.08, 0.07] | 0.04 | -.01 | -0.32 | .748 |
|  | **gender** |  |  | .01 | 0.14 | .892 |
|  | male | *ref* |  |  |  |  |
|  | female | 0.43 [-1.85, 2.72] | 1.16 |  |  |  |
|  | diverse | -0.77 [-7.69, 6.15] | 3.51 |  |  |  |
|  | **employment status** |  |  | -.03 | -0.71 | .479 |
|  | employed | *ref* |  |  |  |  |
|  | unemployed | 0.20 [-1.88, 2.29] | 1.06 |  |  |  |
|  | other | -1.60 [-4.84, 1.63] | 1.65 |  |  |  |
|  | **relationship status** |  |  | .04 | 0.92 | .360 |
|  | single | *ref* |  |  |  |  |
|  | in relationship | 0.93 [-0.98, 2.83] | 0.97 |  |  |  |
|  | **highest level of education** |  |  | .00 | -0.04 | .964 |
|  | none | *ref* |  |  |  |  |
|  | basic or intermediate secondary | -2.42 [-23.87, 19.03] | 10.80 |  |  |  |
|  | vocational | -4.64 [-26.33, 17.06] | 10.92 |  |  |  |
|  | secondary qualifying for university admission | -1.81 [-23.14, 19.52] | 10.74 |  |  |  |
|  | university | -2.24 [-23.56, 19.09] | 10.74 |  |  |  |
|  | other | -5.16 [-27.37, 17.04] | 11.20 |  |  |  |
|  | fear of coronavirus (PAS) | 0.18 [0.01, 0.35] | 0.08 | .10 | 2.30 | **.022** |
|  | *emotion regulation* |  |  |  |  |  |
|  | emotion regulation (DERS) | 0.13 [0.07, 0.18] | 0.03 | .27 | 4.87 | **<.001** |
|  | *emotion regulation strategies* |  |  |  |  |  |
|  | MVPA, accelerometer-measured | -2.08 [-5.02, 0.87] | 1.50 | -.03 | -0.50 | .614 |
|  | LPA, accelerometer-measured | 0.00 [-0.01, 0.01] | 0.01 | -.05 | -1.05 | .296 |
|  | SB, accelerometer-measured | 0.00 [0.00, 0.00] | 0.00 | -.02 | -0.40 | .692 |
|  | PA, self-reported (BSA-F) | -0.49 [-2.19, 1.21] | 0.86 | -.02 | -0.47 | .639 |
|  | MVPA (accelerometer-measured) x  PA-related affect regulation (PA-AR) | 2.48 [-1.53, 6.49] | 2.03 | .06 | 1.31 | .191 |
|  | LPA (accelerometer-measured) x  PA-related affect regulation (PA-AR) | -0.01 [-0.02, 0.01] | 0.01 | -.04 | -0.91 | .363 |
|  | PA self-reported (BSA-F) x  PA-related affect regulation (PA-AR) | 0.42 [-1.93, 2.77] | 1.19 | .01 | 0.32 | .746 |
|  | repetitive negative thinking (PTQ) | 0.27 [0.16, 0.38] | 0.06 | .27 | 4.94 | **<.001** |
|  | sleep quality (PSQI) | 0.76 [0.51, 1.00] | 0.13 | .26 | 6.09 | **<.001** |
|  |  | ***F*(17, 364) = 13.94, *p* < .001*, adj. R^2^* = 0.371** | | | | |
| Incremental validity of model 2 vs. model 3 | | ***F*(10, 17318.12) = 7.23, *p* < .001, *Δ adj. R^2^* = 0.107** | | | | |
| *Note.* B: pooled unstandardized regression coefficient, 95% CI: 95% confident interval for B, SE: standard error, β: pooled standardized regression coefficient. Statistical significance: *p* < .05.  PAS = Pandemic Anxiety Scale (McElroy et al., 2020); DERS = Difficulties in Emotion Regulation Scale (Gratz & Roemer, 2004); PTQ = Perseverative Thinking Questionnaire (Ehring et al., 2011); PSQI = Pittsburgh Sleep Quality Index (Buysse, Reynolds, Monk, Berman, & Kupfer, 1989).  Physical activity (PA; in minutes/week) assessed through accelerometer data and categorized by the metabolic equivalent of tasks (MET) into sedentary behavior (SB; < 2.0 MET), light PA (LPA; 2.0-2.9 MET) and moderate to vigorous PA (MVPA; ≥ 3.0 MET) (Garber et al., 2011) or self-report via the Physical Activity Index and Exercise Index of the Physical Activity, Exercise, and Sport Questionnaire (BSA-F) (Fuchs, Klaperski, Gerber, & Seelig, 2015); PA-AR = PA-related affect regulation assessed with the corresponding subscale of the PA-related health competence questionnaire (Sudeck & Pfeifer, 2016). | | | | | | |
